# Metabolic syndrome associated with the onset of depressive symptoms among women but not men in rural Northeast China

**DOI:** 10.1186/s12888-020-02668-z

**Published:** 2020-05-24

**Authors:** Shasha Yu, Xiaofan Guo, Guang Xiao Li, Hongmei Yang, Liqiang Zheng, Yingxian Sun

**Affiliations:** 1 Department of Cardiology, First Hospital of China Medical University, 155 Nanjing North Street, Heping District, Shenyang, 110001 China; 2 Department of Clinical Epidemiology, Institute of Cardiovascular Diseases, First Hospital of China Medical University, Shenyang, 110001 China; 3 Department of Clinical Epidemiology, Shengjing Hospital of China Medical University, Shenyang, 110004 China

**Keywords:** Major depressive symptom, Gender difference, Incidence, MetS, Metabolic disorders

## Abstract

**Background:**

The present study aimed to assess the cumulative incidence of major depressive disorder (MDD) among rural Chinese residents. Furthermore, we intended to estimate whether metabolic syndrome (MetS) was associated with MDD by both cross-sectional and prospective analysis.

**Method:**

Data of 11,675 residents (46.3% men) was used for cross-sectional analysis. The residents were followed up with median 4.66 years. MDD was diagnosed using the Patient Health Questionnaire-9 (PHQ-9). The data of 2796 individuals without any depressive symptoms was used for prospective analysis.

**Result:**

With median of 4.66 years follow-up, the cumulative incidence of MDD among rural residents was 3.9%. Women had significantly higher cumulative incidence of MDD than men (5.3% for women and 2.9% for men, *P* < 0.01). The incidence of MDD was significantly higher among women with MetS (7.3% vs. 3.8%, *P* < 0.001), hypertriglyceridemia (7.0% vs. 4.5%, *P* < 0.001) or elevated blood pressure (6.4% vs. 3.4%, *P* < 0.001) at baseline compared with those without them. There was no incidence difference of MDD among men with or without baseline metabolic disorders. In prospective study, after adjusting possible confounders, baseline MetS was associated with higher incidence of MDD (OR: 1.82, 95%CI: 1.01, 3.27, *P* = 0.045) in women but not men (OR: 1.84, 95%CI: 0.88, 3.83, *P* = 0.104).

**Conclusion:**

Cumulative incidence of MDD in rural China was higher among women than among men. Baseline MetS was associated with higher cumulative incidence of MDD in women but not men. More concern should be put on women with MetS in case of onset depressive symptom in future.

## Background

Major depressive disorder (MDD) is a common mental disorder and is associated with a higher risk for many comorbid conditions, such as heart failure, cancer and stroke [1]. Metabolic syndrome (MetS) is a cluster of cardio-metabolic risk factors and comorbidities conveying high risk of both cardiovascular events and cerebrovascular disease [2]. Both depression and MetS are responsible for huge socioeconomic costs with their resulting morbidity and mortality worldwide. Furthermore, evidence has confirmed that chronic medical conditions such as cancer, chronic kidney disease, and metabolic risk factors, such as obesity, diabetes, and dyslipidemia, showed comorbidity with depression [3, 4]. Regarding the association between physical conditions and MDD, previous studies have concluded that subjects with one or more metabolic disorders had a higher possibility to develop depression [5–7]. Therefore, it is necessary to estimate the possible relationship between MetS and MDD for better screening and control of both diseases.

Studies defining the association between MetS/metabolic disorders and later onset of depression mostly enrolled subjects from urban or developed areas. Even so, there were inconsistent results. Some claimed that MetS predicted an increased risk for incident depression, while others reported that MetS at bassline did not increase the risk for depression [8, 9]. The conflicting results were attributed to the difference in sample size, study design, and characteristics of the sample. Population heterogeneity plays an important role in the research into MDD [10]. Therefore, it is necessary to evaluate the incidence of MDD and the possible relationship between MDD and MetS in a comprehensive group of general subjects. Accumulating evidence has indicated an increasing trend of depressive symptoms and MetS among subjects from rural areas [11]. Rural areas have their own characteristics, such as lagging behind urban areas in economic development, lower educational levels, and less mental health care and concern about health status, especially mental health. Moreover, a previous study confirmed that lifestyle factors such as frequent alcohol consumption, cigarette smoking, and a sedentary lifestyle with low physical activity correlating with metabolic disorders were relevant to an increased risk for the incidence of depression [12]. People living in rural areas in China have relatively lower educational status and annual income and higher rates of alcohol consumption, smoking, and physical activity intensity due to farm work [13, 14]. These characteristics could affect the association between MDD and MetS among rural subjects. Therefore, it is necessary to evaluate the incidence of MDD and its relationship with MetS among rural residents.

To our knowledge, there is an obvious paucity of studies estimating the association between MetS and MDD. Most of the studies to date are limited by small sample sizes and cross-sectional designs. Due to the advantages and disadvantages of cross-sectional and prospective analyses, we intend to use both analyses to evaluate the possible relationship between MetS and MDD. To the best of our knowledge, only one population cohort study has concurrently investigated the cross-sectional and prospective relationship between MetS and MDD in participants from the same population [15]. However, this study enrolled participants from developed countries. Many previous studies inferred that there is a gender-specific association between MetS and MDD [16, 17]. Kinder et al. enrolled 3186 young men and 3003 women, aged 17–39 years, and claimed that the association between MetS and MDD was found in women but not in men [18]. In contrast, research conducted by Herva, with a mean age of 31 years, found no association between MetS and MDD [19]. There is still a lack of consistent results regarding the relationship between MetS and MDD in the general population. Therefore, we subdivided the total subjects according to gender and to determine whether gender discrepancy existed in the association between MetS and MDD. First, we intended to report the prevalence of MDD among different metabolic disorders at baseline and determine out whether metabolic disorders are associated with MDD in a cross-sectional analysis. Second, we announced the cumulative incidence of MDD at follow-up among subjects without any depressive symptoms at baseline and estimated whether baseline metabolic disorders increase the incidence of MDD in a prospective analysis.

## Method

### Data source and study subjects

The design and inclusion criteria of the community-based prospective cohort study, named the Northeast China Rural Cardiovascular Health Study (NCRCHS), have been described previously [13, 14]. In all, 11,956 participants older than 35 years were enrolled from three countries in LiaoNing Province (Dawa, Zhangwu and Liaoyang) between 2012 and 2013. The Ethics Committee of China Medical University approved this study (Shenyang, China AF-SDP-07-1, 0–01). During 2015 and 2017, we invited participants at baseline to attend the follow-up study. In total, 1256 out of 11,956 subjects were excluded due to a lack of contact information. Ultimately, 10,349 participants finished the follow-up visits (median 4.66 follow-up years). All participants signed the written informed consent. We consider covariables that have complete information from the baseline visit in the present analysis.

### Study population for cross-sectional analysis

**Fig. 1:**
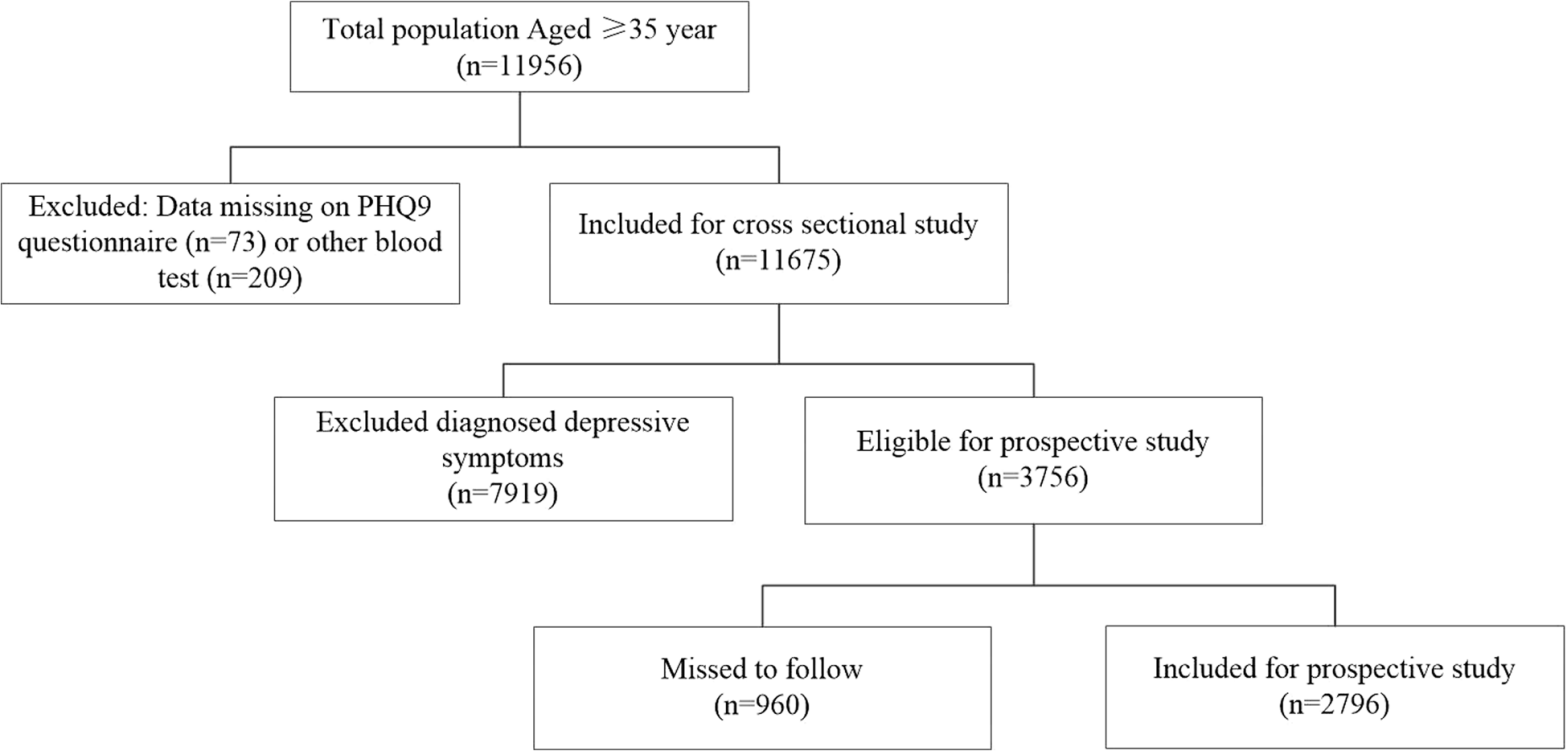
Flow chart for the selection of study subjects for prospective analysis

From a total of 11,956 participants, we excluded those with missing data on serum blood test (209) and those who did not finish the PHQ-9 questionnaire (73), leaving 11,675 participants (5408 men and 6267 women). Among this population, 698 subjects (6%) had severe depressive symptoms (8.1% for women; 3.5% for men) (Fig. 1).

### Study population for prospective analysis

For the purpose of the prospective analysis regarding participants’ future depressive status, we enrolled participants with PHQ score = 0 at baseline. After excluding those lost to follow-up (*n* = 960), 2796 participants (1543 men and 1253 women) were followed until the end of the study (Fig. 1).

### Study variables

At baseline, participants were asked to finish a standardized questionnaire that contained detailed information about socioeconomic factors, lifestyle, demographic characteristics, and chronic disease history. Regular exercise was defined as yes or no. Self-reported history of cerebrovascular diseases, such as ischemic stroke, hemorrhagic stroke, and cardiovascular diseases, such as coronary heart disease and chronic heart failure at baseline, was recorded and confirmed by their medical records. Educational level included ≤ primary school, middle school, and ≥ high school. Annual income of the family was categorized into ≤5000 CNY/year, 5000–20,000 CNY/year and > 20,000 CNY/year. Waist circumference was measured as previously described [13]. Obesity was defined using body mass index (BMI) criteria with the cutoff ≥28 kg/m^2^ [BMI = weight (kg)/height (m) ^2^] [20]. Blood pressure was measured automatically followed the standard criteria using an electronic sphygmomanometer (HEM-907; Omron, Tokyo, Japan). Systolic blood pressure (SBP) more than 140 mmHg and/or diastolic blood pressure (DBP) more than 90 mmHg, with or without medication, were defined as hypertension [21]. After fasting for at least 12 h, participants were gathered together to take blood samples by trained nurses. Fasting plasma glucose (FPG) and lipid profiles, such as low-density lipoprotein cholesterol (LDL-C), high-density lipoprotein cholesterol (HDL-C), total cholesterol and triglyceride, were analyzed enzymatically. The Chronic Kidney Disease Epidemiology Collaboration (CKD-EPI) equation was performed to calculate estimated glomerular filtration rate (eGFR) [22]. MetS was diagnosed with the presence of any 3 of 5 risk factors [23].

Depressive symptoms were diagnosed using the PHQ-9 score. Participants finished the PHQ-9 questionnaire during the baseline and follow-up visits. Each of the nine PHQ depression items corresponds to one of the DSM-IV diagnostic criteria for symptoms for MDD [24]. The specific details of the questionnaire have been described previously [25]. In the present study, PHQ-9 scores ≥10 are diagnosed to be MDD [25].

### Statistical analysis

Mean values ± standard deviations were used to describe continuous variables, and categorical variables were reported as numbers together with percentages. ANOVA, t-test, nonparametric test or the χ2-test were performed to evaluate differences among categories as appropriate. We estimated the cross-sectional and prospective association of MetS with depressive symptoms using logistic regression; we calculated odds ratios (ORs) and 95% confidence intervals (CIs) for depressive symptoms. For these analyses, three models were used. Model one adjusted age. Model two adjusted age, current smoking and drinking, race, educational levels, income level, regular exercise, children number, and chronic diseases (including heart disease, cerebral diseases, and kidney diseases). Model three adjusted age, current smoking and drinking, race, educational levels, income level, regular exercise, children number, chronic diseases (including heart disease, cerebral diseases, and kidney diseases), BMI and eGFR. SPSS version 17.0 software was used to calculate all the statistical analyses, and statistical significance was defined as *P* ≤ 0.05.

## Result

### Prevalence of MDD among participants with or without MetS or other metabolic disorders

**Fig. 2:**
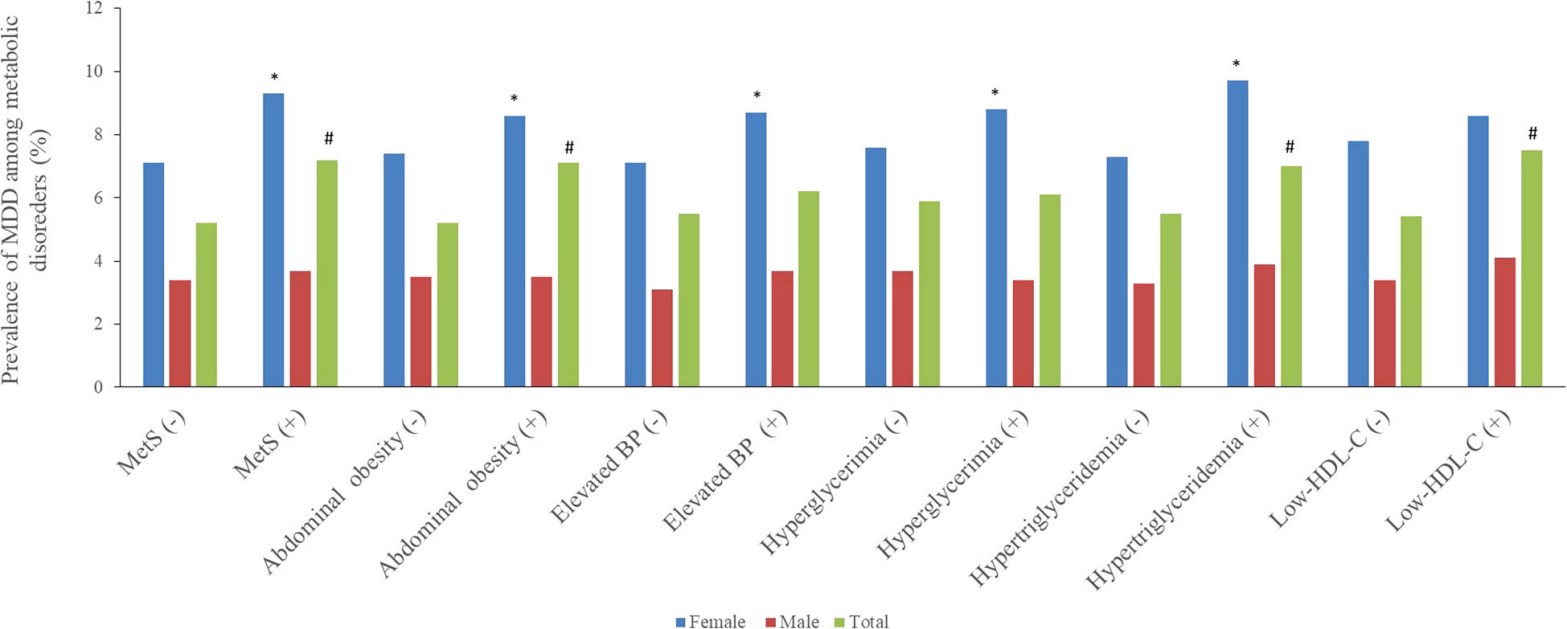
Prevalence of major depressive symptoms among residents with or without MetS and different metabolic disorders. MetS: metabolic syndrome

To evaluate the relationship between different metabolic disorders and MDD, we listed the prevalence of MDD among participants by metabolic disorder status (Fig. 2). In female participants, there were significantly higher prevalence rates of MDD among subjects with MetS (9.3% vs. 7.1%, *P* = 0.001), abdominal obesity (8.6% vs. 7.4%, *P* = 0.045), elevated BP (8.7% vs. 7.1%, *P* = 0.014), hyperglycemia (8.8% vs. 7.6%, *P* = 0.048) and hypertriglyceridemia (9.7% vs. 7.3%, *P* = 0.001) compared to subjects without these metabolic disorders. In men, there were no significant differences in the prevalence of MDD between participants with or without metabolic disorders.

### Cross-sectional analysis of the association between MDD and MetS/metabolic disorders

**Fig. 3:**
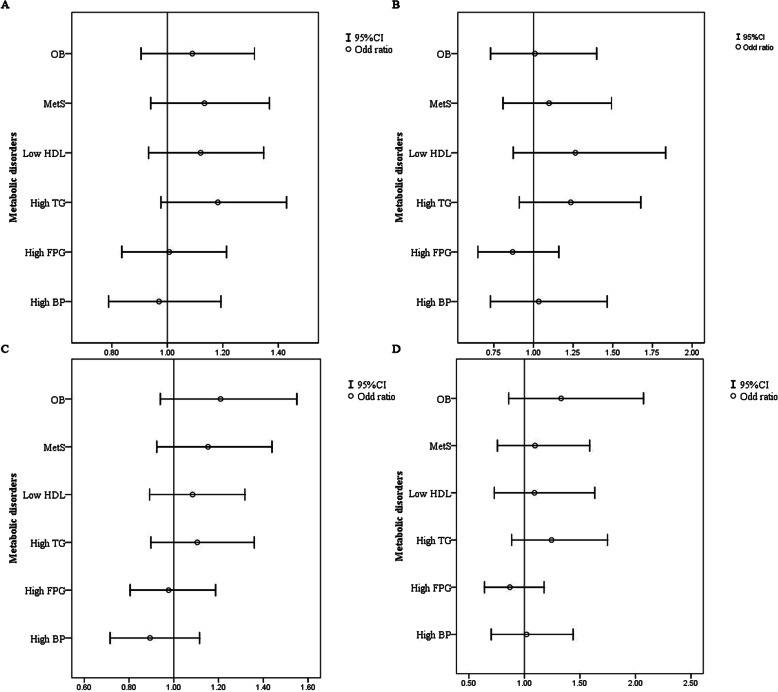
Association between MetS, metabolic disorders and risk of severe depressive symptom among both gender in cross-sectional analysis. **a.b** Adjusted for age, current smoking and drinking, race, educational levels, income level, regular exercise, children number, chronic diseases (including heart disease, cerebral diseases, and kidney diseases) in women and men. **c.d** Except adjusting the confounders in A and B, further adding eGFR and BMI adjusting in both gender

Figure 3 shows the association between MetS, metabolic disorders and risk of MDD among both genders. In Fig. 3.A and B, we can see that, in both women and men, after adjusting for age, current smoking and drinking, race, educational levels, income level, regular exercise, children number, and chronic diseases (including heart disease, cerebral diseases, and kidney diseases), there is lack of a significant relationship between MetS, metabolic disorders and MDD. When we further added eGFR and BMI in to the adjustments, MetS and other metabolic disorders still did not correlate with MDD.

### Baseline characteristics of participants with new-onset MDD

Participants with newly diagnosed MDD were relatively older and were less likely to be current drinkers at baseline (Table 1). Depressive residents were more likely to have regular exercise than nondepressive residents. In addition, participants with newly diagnosed MDD had relatively higher prevalence of primary school or below education level. There were gender differences in baseline characteristics of depressive and nondepressive participants. In men with newly diagnosed MDD, the rate of lower annual income was higher than that in nondepressive participants, a correlation that did not exist among women. Regarding metabolic parameters, only DBP among men showed a significant difference at baseline among depressive and nondepressive participants.

**Table 1:** Baseline characteristics of residents by onset of depressive symptoms by gender

	Total (*n* = 2796)		Men (*n* = 1543)		Women (*n* = 1253)	
	Depressed (*n* = 110)	Not depressed (*n* = 2686)	Depressed (*n* = 66)	Not depressed (*n* = 1187)	Depressed (*n* = 44)	Not depressed (*n* = 1499)
**Age (years)**	**54.56 ± 10.48**	**52.10 ± 10.25**	54.85 ± 11.06	53.04 ± 10.41	**54.38 ± 10.17**	**50.91 ± 9.92**
**Ethnicity (others)**	12 (10.6)	196 (7.2)	4 (9.1)	114 (7.5)	8 (8.9)	82 (6.8)
**Marriage (Yes)**	112 (99.1)	2700 (98.9)	43 (97.7)	1502 (98.3)	69 (100)	1198 (99.8)
**Current smoking status (Yes)**	36 (31.9)	1037 (38.0)	23 (52.3)	877 (57.4)	13 (18.8)	160 (13.3)
**Current drinking status (No)**	**23 (20.4)**	**785 (28.8)**	19 (43.2)	759 (49.7)	4 (5.8)	26 (2.2)
**Regular excising (Yes)**	**32 (29.1)**	**564 (21.0)**	9 (20.5)	294 (19.6)	**23 (34.8)**	**270 (22.7)**
**Education status**						
Primary school or below	**62 (54.9)**	**1156 (42.4)**	19 (43.2)	559 (36.6)	**43 (62.3)**	**597 (49.7)**
Middle school	**36 (31.9)**	**1259 (46.1)**	19 (43.2)	767 (50.2)	**17 (24.6)**	**492 (41.0)**
High school or above	**15 (13.3)**	**314 (11.5)**	6 (13.6)	202 (13.2)	**9 (13.0)**	**112 (9.3)**
**Annual income (CNY/year)**						
≤ 5000	243 (8.9)	9 (8.0)	**7 (15.9)**	**143 (9.4)**	2 (0.2)	100 (7.9)
5000–20,000	1430 (52.4)	68 (60.2)	**30 (68.2)**	**806 (52.8)**	38 (55.1)	624 (52.0)
> 20,000	1055 (38.7)	36 (31.9)	**7 (15.9)**	**578 (37.9)**	29 (42.0)	477 (39.7)
**SBP (mmHg)**	145.65 ± 24.70	142.25 ± 22.48	149.39 ± 24.85	144.70 ± 21.97	143.15 ± 24.48	139.15 ± 22.74
**DBP (mmHg)**	83.92 ± 12.82	82.56 ± 11.71	**88.36 ± 13.70**	**84.20 ± 11.99**	80.95 ± 11.36	80.48 ± 11.00
**BMI (kg/m**^**2**^**)**	25.16 ± 3.62	25.18 ± 3.79	24.66 ± 3.29	25.15 ± 3.51	25.50 ± 3.82	25.22 ± 4.13
**eGFR (mi/min/1.73m**^**2**^**)**	94.20 ± 15.45	95.98 ± 14.17	92.98 ± 16.66	95.84 ± 14.91	96.04 ± 13.39	96.08 ± 13.56
**WC (cm)**	82.88 ± 10.09	83.92 ± 9.94	83.41 ± 9.43	84.58 ± 9.59	82.53 ± 10.57	81.06 ± 10.02
**TC (mmol/L)**	5.26 ± 1.08	5.25 ± 1.09	5.09 ± 0.96	5.26 ± 1.06	5.36 ± 1.15	5.24 ± 1.13
**TG (mmol/L)**	1.73 ± 1.35	1.65 ± 1.62	1.63 ± 1.50	1.71 ± 1.79	1.79 ± 1.26	1.56 ± 1.37
**LDL-C (mmol/L)**	3.01 ± 0.87	2.96 ± 00.83	2.87 ± 0.83	2.97 ± 0.81	3.11 ± 0.88	2.96 ± 0.86
**HDL-C (mmol/L)**	1.43 ± 0.38	1.44 ± 0.41	1.444 ± 0.43	1.44 ± 0.45	1.42 ± 0.34	1.43 ± 0.35
**FPG (mmol/L)**	5.78 ± 1.26	5.89 ± 1.68	5.80 ± 1.40	5.95 ± 1.68	5.78 ± 1.18	5.81 ± 1.68

Data are expressed as the mean ± SD or as n (%)

Abbreviations: *BMI* body mass index, *WC* waist circumference, *CNY* China Yuan (1CNY = 0.161 USD), *SBP* systolic blood pressure, *DBP* diastolic blood pressure, *TG* triglyceride, *LDL-C* low-density lipoprotein cholesterol, *HDL-C* high-density lipoprotein cholesterol, *FPG* fasting plasma glucose. ^a^ Including some ethnic minorities in China, such as Mongol and Manchu

### Prospective analysis of the association between MDD and MetS/metabolic disorders

Overall, 66 new cases of MDD were identified after a median follow-up of 4.66 years. The cumulative incidence of MDD was 3.9% (5.3% for women and 2.9% for men). As shown in Table 2, the incidence rate of MDD was higher in MetS among women (7.3% vs. 3.8%, *P* < 0.001) but not men (3.4% vs. 2.5%, *P* > 0.05). Moreover, women with baseline hypertriglyceridemia (7.0% vs. 4.5%, *P* < 0.001) and elevated BP (6.4% vs. 3.4%, *P* < 0.001) also had higher incidence of MDD compared with those without it. In newly diagnosed MDD, the average number of cases of metabolic disorder was significantly greater than participants without MDD (2.68 ± 1.46 vs. 2.22 ± 1.40, *P* < 0.001). Among men, no significant differences existed in different MetS and other metabolic disorders. Table 2 also shows the different adjusted confounders for MetS and metabolic disorders in both genders. After possible adjustment, in women only, MetS was associated with an increasing incidence of MDD.

**Table 2:** Cumulative incidence of depressive symptoms in relation to MetS and metabolic disorders with a median follow-up of 4.3 years

		Numbers	No. of Depressive incidents	Model one ^a^		Model two ^b^		Model three ^c^	
				OR(95%CI)	*P*-value	OR(95%CI)	P-value	OR(95%CI)	P-value
Women	Mets (−)	**717 (57.2)**	**27 (3.8)**	**1.00(reference)**	**0.032**	**1.00(reference)**	**0.036**	**1.00(reference)**	**0.045**
	MetS (+)	**536 (42.8)**	39 (7.3)*	**1.77 (1.05,2.98)**		**1.76 (1.04,2.98)**		**1.82 (1.01,3.27)**	
	Elevated WC (−)	575 (45.9)	25 (4.3)	1.00(reference)	0.306	1.00(reference)	0.338	1.00(reference)	0.42
	Elevated WC(+)	678 (54.1)	41 (6.0)	1.31 (0.78,2.19)		1.29 (0.77,2.17)		1.31 (0.68,2.50)	
	Hypertriglyceridemia (−)	**883 (70.5)**	**40 (4.5)**	1.00(reference)	0.195	1.00(reference)	0.304	1.00(reference)	0.384
	Hypertriglyceridemia (+)	**370 (29.5)**	26 (7.0)*	1.41 (0.84,2.37)		1.32 (0.78,2.24)		1.28 (0.74,2.21)	
	Low HDL-C (−)	788 (62.9)	39 (4.9)	1.00(reference)	0.573	1.00(reference)	0.576	1.00(reference)	0.574
	Low HDL-C (+)	465 (37.1)	27 (5.8)	1.16 (0.70,1.92)		1.16 (0.69,1.94)		1.16 (0.69,1.97)	
	Hyperglycemia (−)	733 (58.5)	33 (4.5)	1.00(reference)	0.343	1.00(reference)	0.383	1.00(reference)	0.458
	Hyperglycemia (+)	520 (41.5)	33 (6.3)	1.28 (0.77,2.12)		1.26 (0.75,2.12)		1.22 (0.72,2.08)	
	Elevated BP (−)	471 (37.6)	16 (3.4)	1.00(reference)	0.096	1.00(reference)	0.089	1.00(reference)	0.127
	Elevated BP (+)	**782 (62.4)**	50 (6.4)*	1.66 (0.91,3.02)		1.69 (0.92,3.09)		1.62 (0.87,3.01)	
Men	Mets (−)	1021 (66.2)	26 (2.5)	1.00(reference)	0.302	1.00(reference)		1.00(reference)	
	MetS (+)	522 (33.8)	18 (3.4)	1.38 (0.75,2.54)		1.46 (0.78,2.70)	0.234	1.84 (0.88,3.83)	0.104
	Elevated WC (−)	1076 (69.7)	29 (2.7)	1.00(reference)	0.515	1.00(reference)	0.276	1.00(reference)	0.102
	Elevated WC(+)	467 (30.3)	15 (3.2)	1.23 (0.65,2.33)		0.71 (0.38,1.32)		2.06 (0.87,4.90)	
	Hypertriglyceridemia (−)	1027 (66.6)	32 (3.2)	1.00(reference)	0.428	1.00(reference)	0.421	1.00(reference)	0.392
	Hypertriglyceridemia (+)	516 (33.4)	12 (2.3)	0.76 (0.39,1.49)		0.76 (0.38,1.49)		0.73(.35,1.51)	
	Low HDL-C (−)	1310 (84.9)	36 (2.7)	1.00(reference)	0.503	1.00(reference)	0.545	1.00(reference)	0.436
	Low HDL-C (+)	233 (15.1)	8 (3.4)	1.31 (0.60,2.86)		1.28 (0.58,2.84)		1.39 (0.61,3.20)	
	Hyperglycemia (−)	750 (48.6)	25 (3.3)	1.00(reference)	0.231	1.00(reference)	0.402	1.00(reference)	0.307
	Hyperglycemia (+)	793 (51.4)	19 (2.4)	0.69 (0.38,1.27)		1.32 (0.69,2.52)		0.72 (0.39,1.35)	
	Elevated BP (−)	386 (25.0)	11 (2.8)	1.00(reference)	0.812	1.00(reference)	0.897	1.00(reference)	0.826
	Elevated BP (+)	1157 (75.0)	33 (2.9)	0.92 (0.45,1.86)		0.95 (0.47,1.96)		0.92 (0.44,1.94)	

^a^adjusted for age; ^b^adjusted for age, current smoking and drinking, race, educational levels, income level, regular exercise, children number, chronic diseases (including heart disease, cerebral diseases, kidney diseases); ^c^adjusted for age, current smoking and drinking, race, educational levels, income level, regular exercise, children number, chronic diseases (including heart disease, cerebral diseases, kidney diseases), BMI and eGFR

## Discussion

The present study estimated the prevalence of MDD among rural residents with or without MetS or other metabolic disorders and found that MDD was more prevalent among women but not men with MetS and other metabolic disorders, except for low HDL-C, compared with those without them. However, after adjusting for possible confounders, MetS or other metabolic disorders did not associate with MDD in cross-sectional analysis. We further focused on residents without any depressive symptoms at baseline and found that during the follow-up, the cumulative incidence of MDD was higher in MetS among women only. Further adjusted analysis revealed that baseline MetS rather than other metabolic disorders was associated with an increasing incidence of MDD. This relationship only existed among women; it was not detected among men.

The rate of MDD varies from country to country. Recently, a global view of depression reported prevalence rates of depression around the world, such as 22.5% in Afghanistan, 6.16% in Switzerland, 4.45% in United States and 3.02% in China [26]. However, in our cross-sectional analysis, the prevalence of severe depressive symptoms was 6.0% (8.1% for women and 3.1% for men, *P* < 0.001), which was relatively higher than previously reported. Moreover, both the prevalence and cumulative incidence of MDD were significantly higher among rural women than men, which was consistent with previous studies [27]. As a major public health problem, depression has been proved to have a deteriorating effect on health and to coexist with chronic diseases. The incidence of depression increased among subjects with hypertension, coronary heart diseases or diabetes, and both the treatment and prognosis can be affected by these chronic diseases [28]. In our study, women had significantly higher rates of chronic medical diseases than men at baseline, which might partially account for why MDDs were more prevalent among rural women (28.9% vs. 21.9%, *P* < 0.001). In addition to the biological difference that might account for this variation, many biopsychosocial factors are responsible for this discrepancy. For instance, a number of prospective observational cohort studies claimed that mental disorders have a close relationship with poor socioeconomic factors, such as lower income and joblessness [29]. The National Health and Nutrition Examination Survey (NHANES) cohorts conducted in the U.S. also confirmed that as the educational level increased, the rate of MDD decreased significantly [30]. In our cross-sectional analysis, we found that relatively higher educational level (Middle school: OR: 0.78; 95%CI: 0.65, 0.95; High school or above: OR: 0.55; 95%CI: 0.38, 0.80) was significantly associated with MDD. In rural participants, women had apparently lower rates of high educational status than men (Middle school: 35.3% vs. 46.6%; High school or above: 8.1% vs. 11.9%; all *P* < 0.05). The present study further confirmed that there were many other biopsychosocial factors that might affect the risk of depression.

In recent years, the prevalence of MetS and metabolic disorder among rural residents was almost comparable to that among urban citizens [31, 32]. Estimations of the association between MetS and depressive symptoms is needed urgently. In our study, we found a significantly higher rate of MDD among women with MetS or other metabolic disorders, except for low HDL-C. This relationship did not exist among men, which might partially be due to the relatively lower prevalence of MDD among men. However, when we adjusted the possible confounders in the cross-sectional analysis, MetS and metabolic disorders were not significantly associated with a higher risk of MDD among either women or men. Hence, to evaluate this association better, we conducted a prospective analysis. During the median 4.3 years of follow-up, the cumulative incidence of MDD among rural residents was 3.9% (5.3% for women and 2.9% for men). Our data were close to those of a prospective study conducted in central Kazakhstan, which reported rates of 28.51% with a minimal degree of depressive symptoms, 27.7% with mid, 13.7% with moderate, 4.6 and 1.2% with severe and very severe degrees of symptoms [33]. In women with baseline MetS, the cumulative incidence was significantly higher than among women without MetS. Similar findings were observed in women with either hypertriglyceridemia or elevated blood pressure. After adjusting for possible confounders, only MetS exhibited a meaningful relationship with MDD. Many epidemiological studies have consistently confirmed a coexistence of depression with MetS [4]. However, until recently, the exact mechanisms linking MetS to depression have been unclear. However, some hypotheses had been formulated. First, recent studies uncovered that both MetS and depression are associated with chronic, low-grade inflammation, which is characterized by elevated levels of circulating proinflammatory cytokines, alteration of leukocyte population frequencies in the blood and accumulation of immune cells in tissues such as the brain [34]. During this chronic oxidative and inflammatory stress, these cytokines induce depressive-like behavior through interruptions of neurotransmitter synthesis and signal transduction [35]. Second, there is a hypothesis that alteration of the gut microbiota might affect mental disorders [36]. Obesity is always accompanied by insulin resistance and can easily progress into MetS. High-fat diet, and diets containing more meat and fewer vegetables were prevalent among MetS participants. Hence, the turbulence of gut microbiota might alter the psychological state, suggesting that diet changes could be a useful strategy to control psychological disorders. However, more studies are needed to understand the possible effect of gut probiotics on mental health [36]. Third, a previous study inferred that peripheral hormones such as leptin and ghrelin might have an effect on mood regulation [4]. Diet patterns and physical activity play roles in both MetS and depression. One useful strategy might be lifestyle modification, such as regular exercise and a healthier diet pattern, such as the Mediterranean diet. Studies have reported that exercise therapy can improve both mental and physical health in subjects with major depression, while the Mediterranean diet may contribute to the prevention of a series of mental disorders [37, 38].

One interesting finding in the present study is that there were different results between the cross-sectional and prospective analyses. Baseline MetS was associated with a higher incidence of depressive symptoms in the prospective analysis, but not with a higher prevalence of depressive symptoms in the cross-sectional analysis. We further examined the data and found out that in the cross-sectional analysis, possible confounders such as increased age (OR = 1.02, 95%CI = 1.01, 1.03), higher annual family income (OR = 1.35, 95%CI = 1.18, 1.55), and chronic medical diseases (OR = 1.81, 95%CI = 1.57, 2.23) were relevant to higher prevalence rates of depressive symptoms, whereas higher educational level (OR = 0.79, 95%CI = 0.68, 0.92) and regular exercise (OR = 0.70, 95%CI = 0.56, 0.87) protected subjects from depressive symptoms. However, in the prospective analysis, except for chronic medical disease (OR = 1.75, 95%CI = 1.01, 3.05) and baseline MetS, none of the confounders mentioned above was associated with the incidence of depressive symptoms. Therefore, these findings might be due to the possible effects of lifestyle and socioeconomic factors that influence the association between MetS and depressive symptoms, which had been well proved previously [12].

Another interesting finding of the present study was the gender discrepancy in the association between MetS and depressive symptoms. In accordance with our findings, Sharon Toker and colleagues reported that, among apparently healthy employed men and women, depression among women, but not men, was associated with MetS [39]. One possible reason might be due to the relatively higher incidence of depressive symptoms among women. There is a sex difference in the expression of depression. Women are more expressive of their fearful and sad feelings than are men [40]. Another possible reason might be the different impact of sex hormones on depressive symptoms. A previous study confirmed that hormone levels and metabolic parameters were important factors mediating depressive symptoms and cognitive function in women [41]. Further studies are required to better explain this discrepancy.

The present study has some limitations. First, we used the PHQ-9 questionnaire to evaluate the depressive symptoms, which has been used by many epidemiological studies. However, we did not use a comprehensive psychiatric evaluation; thus, we are unable to make a definitive diagnosis of depression. Second, the present study aimed to estimate the cumulative incidence of MDD among rural residents without any depressive symptoms at baseline (means PHQ9 score = 0); therefore, the onset number of severely hypertensive residents was small, which cause bias. Finally, 960 subjects missed the follow-up in the present study. It is possible that the missing data in the categories of MetS and MDD may cause bias in our results.

## Conclusion

In conclusion, even though there was a significantly higher rate of MDD among women residents with MetS/metabolic disorders, after adjusting for confounders, neither MetS nor metabolic disorders were associated with MDD in the cross-sectional analysis. Nevertheless, in a prospective analysis, baseline MetS in women was associated with a higher incidence of MDD. This suggested that more concern should be paid to the mental health of women with MetS from rural areas of China.

## Data Availability

Enquiries regarding the availability of primary data should be directed to the principal investigator Professor Yingxian Sun (sunyingxiancmu1h@163.com).

## Abbreviations

MDD: Major depressive disorder
MetS: Metabolic syndrome
PHQ-9: Patient Health Questionnaire-9
BMI: Body mass index
SBP: Systolic blood pressure
DBP: Diastolic blood pressure
FPG: Fasting plasma glucose
CKD-EPI: Chronic Kidney Disease Epidemiology Collaboration equation
eGFR: Estimated glomerular filtration rate
LDL-C: Low-density lipoprotein cholesterol
HDL-C: High-density lipoprotein cholesterol
